# Delayed phrenic nerve injury after cryoballoon ablation for atrial fibrillation

**DOI:** 10.1016/j.hrcr.2025.05.011

**Published:** 2025-05-14

**Authors:** Yu-Shan Huang, Fa-Po Chung

**Affiliations:** 1 Division of Cardiology, Department of Medicine, Taichung Veterans General Hospital, Taipei, Taiwan; 2 Institute of Clinical Medicine and Cardiovascular Research Center, National Yang Ming Chiao Tung University, Taipei, Taiwan; 3 Division of Cardiology, Department of Medicine, Taipei Veterans General Hospital, Taipei, Taiwan

**Keywords:** Atrial fibrillation, Pulmonary vein isolation, Cryoballoon ablation, Phrenic nerve palsy, Delayed phrenic nerve injury

**Key Teaching Points**

• Maintaining vigilance during ablation is essential, with the procedure halted immediately upon any loss of phrenic nerve capture, given that phrenic nerve injury generally occurs promptly owing to direct thermal damage.
• Early detection and termination of ablation can prevent phrenic nerve palsy. We can use compound motor action potential or diaphragm motion sensors to assist in this process.
• Delayed phrenic nerve dysfunction can occur beyond the expected timeframe for thermal injury, even if nerve function initially seems to have recovered at the end of the procedure.

## Introduction

Cryoballoons have proven to be effective for pulmonary vein isolation (PVI) in patients with drug-refractory paroxysmal atrial fibrillation (AF). The efficacy and safety of cryoablation are similar to those of radiofrequency ablation (RFA), but the procedure time is shorter.^1^, ^2^, ^3^ The most common complication associated with cryoballoon ablation (CBA) is phrenic nerve palsy (PNP) with hemidiaphragmatic paralysis, occurring in 3% to 11% of clinical procedures.^3^, ^4^, ^5^ In contrast, phrenic nerve injury (PNI) is rarely reported as a complication during RFA, especially when a wide area circumferential ablation is used.^3^ There is no reliable method to predict PNI before the procedure; however, early detection of PNI during the procedure through rigorous monitoring of nerve function and immediate termination of ablation remains the cornerstone of preventing PNP.^6^^,^^7^ PNI has previously been demonstrated as an immediate effect of cryoablation, with loss of diaphragmatic contraction detection. In some cases, this effect has been reported to occur transiently, with recovery within minutes and resumption of diaphragmatic contraction before the end of the procedure.^6^^,^^8^

Even though phrenic nerve function returned rapidly during CBA, this report describes a case of delayed development of PNI beyond the expected time course.

## Case Report

**Figure 1:**
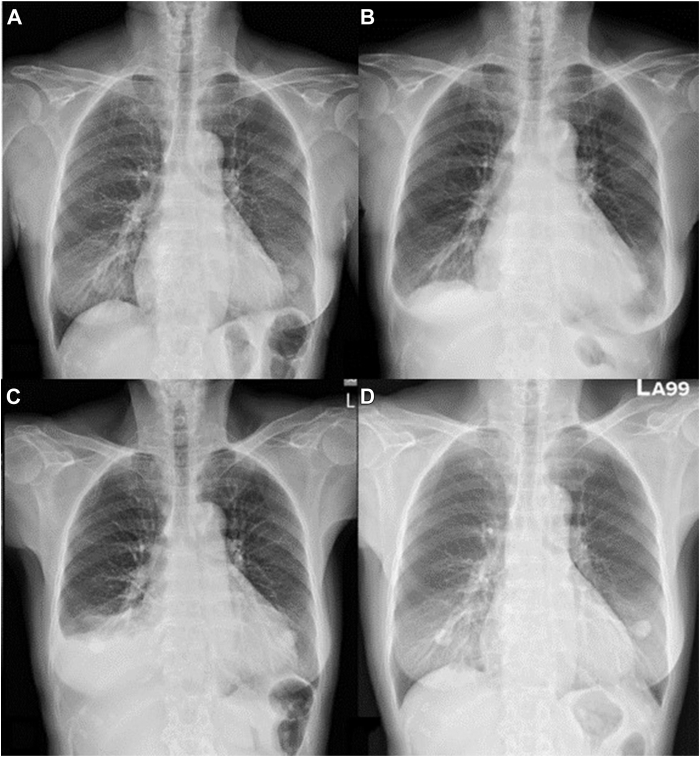
**A:** Posteroanterior chest radiograph before the procedure. **B:** Four hours after the procedure, there was no evidence of unilateral diaphragmatic elevation. **C:** Two weeks after cryoablation showing elevated right diaphragm. **D:** Two months after cryoablation showing recovery of the elevated right diaphragm.

A 72-year-old woman with a 5-year history of AF experienced palpitations and shortness of breath. Despite taking edoxaban 30 mg for anticoagulation (CHA_2_DS_2_-VASc score 2) and flecainide, she remained symptomatic. An echocardiogram revealed a normal left atrium size (anteroposterior diameter, 33 mm) with no significant structural heart disease, and a chest radiograph (CXR) showed no remarkable abnormalities (Figure 1A). After a thorough discussion, transcatheter CBA was recommended.

The preprocedural examination and preparation for CBA have been described previously (37044211). In brief, after general anesthesia and placement of a coronary sinus catheter, a single transseptal puncture was performed via an 8.5F sheath (SL 1, St. Jude Medical), which was subsequently replaced by a 16F deflectable sheath (Polarsheath; Boston Scientific Corporation, Marlborough, MA). PVI was conducted using a novel size-adjustable cryoballoon system (POLARx Fit; Boston Scientific Corporation, Marlborough, MA). Grade 3 or 4 occlusion was intended to be achieved under fluoroscopic guidance.

Cryoablations were initiated from the left pulmonary veins with nadir temperatures of −61 °C and −48 °C and time to isolation (TTI) of 54 seconds and 34 seconds for the left superior pulmonary vein and left inferior pulmonary vein, respectively. To prevent PNI, CBA of the right pulmonary veins was monitored by the diaphragm movement sensor (DMS) under continuous phrenic stimulation using a decapolar catheter.

**Figure 2:**
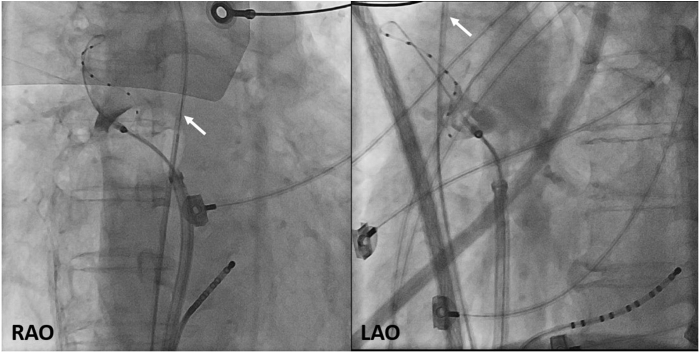
RAO and LAO radiographs showed grade 4 occlusion of the right superior pulmonary vein during cryoballoon ablation. The pacing catheter (*white arrow*) is positioned deep in the superior vena cava at a site where consistent phrenic nerve stimulation is achieved. LAO = left anterior oblique; RAO = right anterior oblique.

During the cryoablation of the right superior pulmonary vein (RSPV) with grade 4 occlusion (Figure 2), isolation was achieved with a TTI of 27 seconds and a nadir temperature of −55 °C. However, CBA was prematurely terminated at 50 seconds owing to a decrease in DMS (from 95% to 70%), indicating PNI. After diaphragm motion recovered within 1 minute, suggesting a transient PNI, right inferior PVI was achieved with a nadir temperature of −56 °C without identifiable TTI. Two applications of CBA with a total duration of 240 seconds were performed.

Given that PNI occurred during the initial application of CBA for the RSPV with a 28 mm cryoballoon, we expanded the cryoballoon to 31 mm for additional RSPV ablation, achieving a nadir temperature of −56 °C. Four PV isolations were finally confirmed, and echocardiography at the end of the procedure revealed no pericardial effusion. The routine CXR taken approximately 4 hours after the procedure showed slight blunting of the bilateral costophrenic angles without unilateral diaphragmatic elevation (Figure 1B). The patient was discharged under stable conditions without immediate sequelae. Two weeks after discharge, the patient experienced worsening dyspnea.

**Figure 3:**
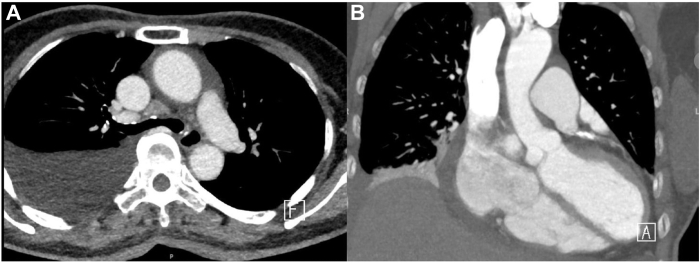
Axial (**A**) and coronal (**B**) chest computed tomography images obtained 2 weeks after cryoablation reveal an elevated right diaphragm and pleural effusion.

An electrocardiogram revealed recurrent AF, and an echocardiogram showed a small amount of pericardial effusion. A CXR indicated an elevated right hemidiaphragm with blunting of the right costophrenic angle (Figure 1C), whereas a follow-up chest computed tomography revealed the right pleural effusion and an elevated right hemidiaphragm (Figure 3), suggesting right PNP.

During hospitalization, the patient underwent cardioversion to restore sinus rhythm. After supportive treatment, she did not report any cough or hiccups and did not require noninvasive mechanical ventilation. The patient was discharged and scheduled for further follow-up to monitor the recovery course of the PNI.

At a follow-up appointment 2 months later, she reported significant improvement in her symptoms, including reduced shortness of breath. A subsequent CXR (Figure 1D) showed significant improvement in the right pleural effusion and elevated right hemidiaphragm, indicating recovery from right PNP. After a blanking period, no further AF recurrences were noted during the 1-year follow-up period after the ablation.

## Discussion

Cryoballoon-based PVI is effective in achieving sustained PVI, offering shorter procedure times than conventional RFA.^1^ However, the most common complication, PNP, occurs in 3% to 11% of cases,^3^^,^^4^^,^^8^ and early detection and termination of CBA are key to preventing this outcome.^3^ In the case presented, PNP developed later despite the early termination of ablation when reduced DMS was detected through continuous phrenic nerve pacing and immediate recovery of transient PNI, highlighting the importance of continuous monitoring of phrenic nerve function once transient PNI was found during CBA.

### Diagnosis of PNI

PNI should be suspected when a patient experiences shortness of breath, often described as difficulty in taking a deep breath, after ablation.

PNI may be suspected when a patient experiences shortness of breath.^9^ This injury can be diagnosed through a clinical examination and a CXR, with the characteristic finding being the elevation of the affected hemidiaphragm. Approximately 90% of unilateral diaphragm palsies are identified by an elevated hemidiaphragm on routine CXR, as shown in our case. If there is any uncertainty, a sniff test with cine fluoroscopy should be performed of the paralyzed hemidiaphragm during inspiration.^10^ The diagnosis should be confirmed with a phrenic nerve conduction study and diaphragm electromyography.

### Monitor phrenic nerve function

Currently, there is no reliable method to predict PNI before the procedure. Monitoring phrenic nerve function is most often performed by palpating the strength of diaphragmatic movements below the costal margin during phrenic nerve pacing. A decrease in the strength of diaphragmatic contraction may indicate PNI; however, variations in respiratory strength of diaphragmatic contraction may sometimes be misinterpreted as PNI.^1^

In addition to abdominal palpation, which is widely regarded as the gold standard, other methods have been introduced. During phrenic nerve pacing, the diaphragmatic compound motor action potential (CMAP) can be reliably recorded using surface electrodes. This method provides valuable information about phrenic nerve function.^8^, ^9^, ^10^ The CMAP amplitude is measured from peak to peak with each phrenic nerve capture, and real-time CMAP amplitudes during ablation are compared with the baseline. Studies showed that a decrease in CMAP amplitude of 35% or more can predict PNI.^10^ Thus, it is recommended to stop ablation if the observed CMAP amplitude decreases by 30% or more from baseline.

In addition to CMAP, the DMS is a new tool for diaphragm monitoring integrated into the console. This sensor connects to an adhesive electrocardiogram electrode placed under the patient’s right costal margin and links to the console. The sensor detects changes in diaphragmatic motion, enabling early identification of PNI, with a 30% threshold reduction, as commonly recommended and adopted from the cutoff used for CMAP. However, in practice, the DMS yielded erroneous diagnoses in 52.4% of cryoapplications, with specificity even lower in patients exhibiting movement during cryoapplications.^11^ Some validation methods should be considered to comprehensively assess the full recovery of phrenic nerve function if false detection of nerve dysfunction occurs with DMS. First, concurrent diaphragmatic CMAP should demonstrate a return of stable, high-amplitude signals without significant latency changes. Second, fluoroscopy, which is more feasible, can visually confirm diaphragmatic movement in real time, independent of the DMS system. Third, if phrenic nerve recovery is suspected, systematic low-output pacing at different sites can help confirm whether the nerve has truly regained function. Further studies are needed to establish a more reliable cutoff for the DMS.

### Clinical course and prognosis of PNI

Currently, there is no straightforward method for restoring PNI or impaired diaphragmatic function. Early detection of PNI and prompt cessation of CBA are crucial to prevent PNP. If phrenic nerve function resumes within a few minutes, cautiously attempting ablation with the balloon positioned more antrally may be considered. If phrenic nerve function does not return promptly, the symptoms of PNI can range from being asymptomatic to exhibiting cough, dyspnea, or severe respiratory complications.^12^ Generally, the prognosis for PNI is favorable, with recovery depending on the extent of the damage and the time required for nerve regeneration. Most cases resolve spontaneously within 3 to 12 months, although rarely, the injury might be permanent. Typically, most PNI recoveries occur within a year.^13^^,^^14^

## Conclusion

It is imperative to remain vigilant and immediately stop the ablation if there is any loss of phrenic nerve capture. Typically, PNI has been described as an immediate result of direct thermal damage leading to nerve dysfunction. However, our case highlighted the occurrence of delayed phrenic nerve dysfunction, which developed beyond the anticipated timeframe, caused by direct thermal injury, despite the phrenic nerve function appearing recovered at the end of the procedure.

## Disclosures

The authors have no conflicts of interest to disclose.
